# Correction: Chronic exercise effects on overall depression severity and distinct depressive symptoms in older adults: A protocol of a systematic and meta-analytic review

**DOI:** 10.1371/journal.pone.0337031

**Published:** 2025-11-17

**Authors:** Melanie Mack, Andreea Badache, Arzu Erden, Christoforos D. Giannaki, Sandra Haider, Antonia Kaltsatou, Burcu Kömürcü Akik, Yaël Netz, Iuliia Pavlova, Pinelopi S. Stavrinou, Claudia Voelcker-Rehage, Michel Audiffren, on behalf of the PhysAgeNet

The Acknowledgment statement for this article is incorrect. The correct statement is: This publication is based upon work from COST Action CA20104 – Network on evidence-based physical activity in old age (PhsAgeNet), supported by COST (European Cooperation in Science and Technology). The complete membership list of the consortium is available here: https://www.cost.eu/actions/CA20104/#tabs+Name:Working%20Groups%20and%20Membership.
