# Supplementary material for: Chronic exercise effects on overall depression severity and distinct depressive symptoms in older adults: A protocol of a systematic and meta-analytic review
Source: PLoS One. 2024 May 23;19(5):e0297348. doi: 10.1371/journal.pone.0297348 (PMC11115242; doi:10.1371/journal.pone.0297348)
Supplement: S1 File — (PDF) [file pone.0297348.s002.pdf]

Search on Web on Science

(1) TS=depress* OR TS=dysthymi* OR TS=(mood NEAR/1 disorder)

(2) TS=random* OR TS=RCT OR TS=(clinical NEAR/1 trial) OR TS=(intervention* NEAR/1 study)

(3) TS=exercise OR TS=(physical NEAR/1 activity) OR TS=(physical NEAR/1 exertion) OR TS=swim* OR TS=gym* OR TS=walk* OR TS=danc* OR TS=jog* OR TS=run* OR TS=cycl* OR TS=bicycl* OR TS=hiking OR TS=(tai NEAR/1 ji) OR TS=(tai NEAR/1 chi) OR TS=yoga OR TS=(qi NEAR/1 gong) OR TS=sport* OR TS=(physical NEAR/1 training) OR TS=(strength NEAR/1 training) OR TS=(weight NEAR/1 training) OR TS=(resistance NEAR/1 training) OR TS=(balance NEAR/1 training) OR TS=(aerobic NEAR/1 training) OR TS=(anaerobic NEAR/1 training) OR TS=(endurance NEAR/1 training) OR TS=(muscle NEAR/1 training) OR TS=exergam* OR TS=(active NEAR/1 video NEAR/1 game) OR TS=Wii OR TS=Kinect OR TS=pilates OR TS=feldenkrais OR TS=(motor NEAR/1 activity) OR TS=(cardiac NEAR/1 rehabilitation)

(4) TS=elder* OR TS=aging OR TS=senior* OR TS=(older NEAR/1 adults) OR TS=(older NEAR/1 patients) OR TS=(older NEAR/1 women) OR TS=(older NEAR/1 people) OR TS=(older NEAR/1 persons) OR TS=(older NEAR/1 subjects) OR TS=(old* NEAR/1 age) OR TS=geriatric* OR TS=gerontolog* OR TS=(late NEAR/1 life) OR TS=(postmenopausal NEAR/1 women) OR TS=Alzheimer* OR TS=Parkinson* OR TS=(mean NEAR/1 age NEAR/1 of NEAR/1 60) OR TS=(mean NEAR/1 age NEAR/1 of NEAR/1 61) OR TS=(mean NEAR/1 age NEAR/1 of NEAR/1 62) OR TS=(mean NEAR/1 age NEAR/1 of NEAR/1 63) OR TS=(mean NEAR/1 age NEAR/1 of NEAR/1 64) OR TS=(mean NEAR/1 age NEAR/1 of NEAR/1 65)

(1) AND (2) AND (3) AND (4)

Search in Academic Search Complete, MEDLINE with Full Text, APA PsycInfo, SPORTDiscus with Full Text, CINAHL Complete via EBCSO

(1) TI(depress* OR TI dysthymi* OR TI mood N0 disorder) OR AB(depress* OR dysthymi* OR mood N0 disorder)

(2) TX(random* OR RCT OR (clinical N0 trial) OR (intervention* N0 study) OR random* OR (clinical N0 trial) OR (intervention* N0 study))

(3) AB(exercise OR (physical N0 activity) OR (physical N0 exertion) OR swim* OR gym* OR walk* OR danc* OR jog* OR run* OR cycl* OR bicycl* OR hiking OR (tai N0 ji) OR (tai N0 chi) OR yoga OR qigong OR (qi N0 gong) OR sport* OR (physical N0 training) OR (strength N0 training) OR (weight N0 training) OR (resistance N0 training) OR (balance N0 training) OR (aerobic N0 training) OR (anaerobic N0 training) OR (endurance N0 training) OR (muscle N0 training) OR exergame OR (active N0 video N0 game) OR Wii OR Kinect OR pilates OR feldenkrais OR (motor N0 activity) OR (cardiac N0 rehabilitation)) OR TI(exercise OR (physical N0 activity) OR (physical N0 exertion) OR swim* OR gym* OR walk* OR danc* OR jog* OR run* OR cycl* OR bicycl* OR hiking OR (tai N0 ji) OR (tai N0 chi) OR yoga OR qigong OR (qi N0 gong) OR sport* OR (physical N0 training) OR (strength N0 training) OR (weight N0 training) OR (resistance N0 training) OR (balance N0 training) OR (aerobic N0 training) OR (anaerobic N0 training) OR (endurance N0 training) OR (muscle N0 training) OR exergame OR (active N0 video N0 game) OR Wii OR Kinect OR pilates OR feldenkrais OR (motor N0 activity) OR (cardiac N0 rehabilitation))

(4) TX(elder* OR aging OR senior* OR (older N0 adults) OR (older N0 patients) OR (older N0 women) OR (old* N0 people) OR (older N0 persons) OR (older N0 subjects) OR (old* N0 age) OR geriatric* OR Parkinson* OR gerontolog* OR (late N0 life) OR (postmenopausal N0 women) OR Alzheimer* OR Parkinson* OR (mean N0 age N2 60) OR (mean N0 age N2 61) OR (mean N0 age N2 62) OR (mean N0 age N2 63) OR (mean N0 age N2 64) OR (mean N0 age N2 65))

(1) AND (2) AND (3) AND (4)

Search in Cochrane CENTRAL (in Title Abstract Keyword)

(1) depress* OR dysthymi* OR (mood NEAR/1 disorder)

(2) random* OR RCT OR (clinical NEAR/1 trial) OR (intervention* NEAR/1 study)

(3) exercise OR (physical NEAR/1 activity) OR (physical NEAR/1 exertion) OR swim* OR gym* OR walk* OR danc* OR jog* OR run* OR cycl* OR bicycl* OR hiking OR (tai NEAR/1 ji) OR (tai NEAR/1 chi) OR yoga OR (qi NEAR/1 gong) OR sport* OR (physical NEAR/1 training) OR (strength NEAR/1 training) OR (weight NEAR/1 training) OR (resistance NEAR/1 training) OR (balance NEAR/1 training) OR (aerobic NEAR/1 training) OR (anaerobic NEAR/1 training) OR (endurance NEAR/1 training) OR (muscle NEAR/1 training) OR exergam* OR (active NEAR/1 video NEAR/1 game) OR Wii OR Kinect OR pilates OR feldenkrais OR (motor NEAR/1 activity) OR (cardiac NEAR/1 rehabilitation)

(4) elder* OR aging OR senior* OR (older NEAR/1 adults) OR (older NEAR/1 patients) OR (older NEAR/1 women) OR (older NEAR/1 people) OR (older NEAR/1 persons) OR (older NEAR/1 subjects) OR (old* NEAR/1 age) OR geriatric* OR gerontolog* OR (late NEAR/1 life) OR (postmenopausal NEAR/1 women) OR Alzheimer* OR Parkinson* OR (mean NEAR/1 age NEAR/1 of NEAR/1 60) OR (mean NEAR/1 age NEAR/1 of NEAR/1 61) OR (mean NEAR/1 age NEAR/1 of NEAR/1 62) OR (mean NEAR/1 age NEAR/1 of NEAR/1 63) OR (mean NEAR/1 age NEAR/1 of NEAR/1 64) OR (mean NEAR/1 age NEAR/1 of NEAR/1 65)

(1) AND (2) AND (3) AND (4)
