# Supplementary material for: Chronic exercise effects on overall depression severity and distinct depressive symptoms in older adults: A protocol of a systematic and meta-analytic review
Source: PLoS One. 2024 May 23;19(5):e0297348. doi: 10.1371/journal.pone.0297348 (PMC11115242; doi:10.1371/journal.pone.0297348)
Supplement: Attachment — Submitted filename: Response to reviewers.docx [file pone.0297348.s003.docx]

**Response to reviewers**

**Reviewer #1:** This systematic review protocol is generally well-written. However, there are some topics that I think should be addressed. Also, I failed to understand how this review is different from the already existing ones on the topic. Is it by analyzing symptoms of depression (MDD and/or dysthymia I guess) one by one? It is not clear if this analysis is possible and how it will be performed.

Response: We thank reviewer#1 for his/her comments that allowed us to improve the review protocol and address some important issues. We are particularly grateful for the comment that it does not seem clear how our review differs from those already available. We have added more information to highlight this.

l. 286-291. *“The review will differ from previous reviews in several important ways: (1) by including a large sample of studies with a wide range of individual and exercise characteristics, the chronic effects of exercise on late-life depression will be examined as a function of a large variety of moderators related to these characteristics; (2) The effects of chronic exercise will be examined not only on the level of overall depression severity but on the level of the different symptoms of depression; (3) The results will be corrected for publication bias with different complementary techniques.”*

We further changed the title with regard to this comment.

*“Chronic exercise effects on overall depression severity and distinct depressive symptoms in older adults: A protocol of a systematic and meta-analytic review”*

l. 65-67. Please refer to the year of this estimates.

Response: We thank the reviewer for pointing this out and have added the information:

l. 71-72. *“The average global prevalence of geriatric depression, including estimates assessed between 1994 and 2020, is 31.74%,…”*

l. 69. Is aging associated with lower socioeconomic status?

Response: Yes. The majority of older adults do not work and have fewer options for a continued income. They are at risk for rising costs of living, which may place them at an economic disadvantage and potentially at lower levels of socioeconomic status.

See for example: Ku, I., & Kim, C. O. (2020). Decomposition analyses of the trend in poverty among older adults: the case of South Korea. *The Journals of Gerontology: Series B, 75*(3), 684-693.

l. 79-84. These sentences need to be supported by references.

Response: We agree with the reviewer. We added references to support the specific aspect which differentiate depression with early- and late-onset depression in older adults:

l. 90-94. *“Furthermore, older individuals with recurrent depression since younger age and those with late-life onset seems to have distinct clinical features, risk factors, and treatment responses [9,18]. Compared with older patients with early-onset depression, patients with late-onset depression appear to have a higher prevalence of medical burden and neurological brain disorders [19], more impairment in neuropsychological tests [20] and dysfunctions of several brain regions [9,20].”*

l. 82. What is medical morbidity?

Response: This is a mistake. We meant “morbidity”. Thank you for bringing this to our attention. We have corrected it (l. 90).

l. 91-93. What you mention as adverse events are considered side (secondary) effects. Normally adverse events in depression are suicide death, suicide attempt and relapse.

Response: We agree and have replaced “adverse events” with “side effects” (l. 97).

l. 98. “which can be easily applied independently” what do you mean by this?

Response: We mean that exercise can be performed autonomously without additional costly equipment or external assistance. We have rewritten the sentence.

l. 104-106. *“By the majority of populations, exercise can be performed autonomously without additional costly equipment or external assistance, has no serious side effects, and is accessible to everyone.”*

l. 135-136. I did not understand this sentence.

Response: We have rewritten the sentence and hope that it is now clear what we want to express.

l. 142-143. *“The effect size of exercise interventions is further modulated by frequency, intensity, and duration of exercise sessions [35].”*

l. 136-138. This sentence needs to be supported by references.

Response: Thank you for pointing this out. We added references.

l. 143-146. *“Exercises with a higher intensity might induce higher acute neuroendocrine responses and long-term adaptations in the brain’s neural architecture than exercises with lower intensity [36]. Meaningful responses may be only visible when the exercises are conducted with a certain frequency and duration [37].”*

l. 192-194. This sentence needs to be supported by references.

Response: We agree and added references.

l. 199-200. *“For instance, the physiological response to equivalent ‘dosages’ of exercise differ between males and females [48].”*

l. 209-212. What would be the benefit of doing this? Also, how are you going to discriminate symptom by symptom?

Response: Given the substantial heterogeneity in the clinical presentation of late-life depression, there is a need to customize the exercise interventions to the unique symptom expressions of each patient. The different symptoms of depression will be thereby defined using the criteria delineated by the DSM-5 to make a diagnosis of depression: depressed mood, loss of interest/pleasure, weight loss or gain, insomnia or hypersomnia, psychomotor agitation or retardation, fatigue, feeling worthless or excessive/inappropriate guilt, decreased concentration, thoughts of death/suicide. We added further information to make it clearer:

l. 219-221. *“However, given the substantial heterogeneity in clinical presentation of late-life depression, it underscores the need of customizing the exercise interventions to the unique symptom expressions of each patient.”*

l. 280-284. *“The different symptoms of depression will be thereby defined using the criteria delineated by the DSM-5 to make a diagnosis of depression: depressed mood, loss of interest/pleasure, weight loss or gain, insomnia or hypersomnia, psychomotor agitation or retardation, fatigue, feeling worthless or excessive/inappropriate guilt, decreased concentration, thoughts of death/suicide. Each of these symptoms will be used as secondary outcomes in the present meta-analysis.”*

l. 251-255. How is your review not going to fall in this same issue?

Response: We apologize for not making this clear in the introduction. Unlike other reviews, we include a wide range of individual and exercise characteristics. By analyzing the moderating effects of these variables, we can provide a more detailed picture and will not fall into the same issue. We added further information to make clear how we differ in this regard from other reviews:

l. 260-266. *“The numerous reviews that have already examined the moderating effects of intervention characteristics and individual characteristics have unfortunately found heterogeneous and partly differential results, probably because of their differential inclusion criteria with respect to those characteristics (e.g., only including mind-body interventions vs only aerobic interventions or only including participants with neurodegenerative disease vs only participants with major depressive disorder). Consequently, the moderating role of those characteristics and their interaction is still not fully understood.”*

l. 286-290. *“(1) by including a large sample of studies with a wide range of individual and exercise characteristics, the chronic effects of exercise on late-life depression will be examined as a function of a large variety of moderators related to these characteristics;”*

l. 298-300. By considering mind-body exercises (yoga, tai chi) and exercises with cognitive therapy how are you going to be sure that the effect is due to exercise itself and not the other components of this kind of interventions?

Response: We assume you refer to the sentence “multicomponent exercise (also including cognitive training)”. By including the type of exercise as a moderator variable and assigning yoga to a category other than multi-component exercises, we can distinguish the effects of the different types of interventions. More precisely, six categories will be distinguished: (1) Aerobic exercises, (2) Strength exercises / Resistance exercises, (3) Dance, (4) Balance exercises / Coordinative exercises, (5) Mind-body exercises, (6) Exergames / Video games / Virtual reality, (7) Multi-component exercises. Then a moderator analysis will be conducted on the effect size with “Type of exercise” as moderator.

l. 330. Why are you not using PubMed or Medline or EMBASE? Is it included in any other?

Response: MEDLINE will be included in the set of databases selected for the search. We added this database to the list of databases (l. 350). We did not plan to use EMBASE or PubMed since they focus on biomedical sciences. However, MEDLINE is included in PubMed.

l. 371-374. What is the rational for this? Why don’t you extract both mean changes?

Response: This was a mistake. We will extract the mean changes of all the outcomes. We revised the sentence accordingly:

l. 292-293. *“If an author reported the results of two primary outcome measures meeting our criteria (i.e., mean change in depressive symptoms according to two different measures), we will use both.”*

l. 380. I am not understanding the secondary outcomes section. Are you defining the secondary outcomes that will be extracted (if they appear in the RCT) and the measures accepted by you? E.g., for sleep quality, if it is not assessed by either PSQI or ISI you will not extract that information?

Response: Thank you for that comment. We agree that more information is necessary to understand our process of defining the secondary outcomes. We added additional information:

l. 395-415. *“Secondary outcomes will include symptoms of depression delineated by the DSM-5 to make a diagnosis of depression. For the selection of the outcomes, we have adhered as closely as possible to each of the specific criteria, which are as follows: 1) Depressed mood most of the day, nearly every day, as indicated by either subjective report (e.g., feels sad, empty, hopeless) or observation made by others (e.g., appears tearful); 2) Markedly diminished interest or pleasure in all, or almost all, activities most of the day, nearly every day; 3) Significant weight loss when not dieting or weight gain (e.g., a change of more than 5% of body weight in a month) or decrease or increase in appetite nearly every day; 4) Psychomotor agitation or retardation nearly every day (observable by others, not merely subjective feelings of restlessness or being slowed down); 5) Insomnia or hypersomnia nearly every day; 6) Fatigue or loss of energy nearly every day; 7) Feelings of worthlessness or excessive or inappropriate guilt (which may be delusional) nearly every day (not merely self-reproach or guilt about being sick); 8) Diminished ability to think or concentrate, or indecisiveness, nearly every day; 9) Recurrent thoughts of death (not just fear of dying), recurrent suicidal ideation without a specific plan, or a suicide attempt or a specific plan for committing suicide. We have already performed the preliminary data selection and reviewed all studies to determine how many studies are available reporting results of a valid instrument to measure the symptoms included in the criteria. If several instruments were available to assess a given symptom, we defined an order determined by the validity and frequency of use of the measurement instrument. An exception is the secondary outcome executive functions (see below). Here we will collect all reported measures. If not enough studies (less than 3) report a valid measure for a particular symptom, we will not assess that symptom as secondary outcome. “*

l. 432. What is very light PA?

Response: We mean “light PA”, not “very light PA”. We used categories suggested by the American College of Sports Medicine (ACSM, 2017). The intensity of exercise will be determined by a dual approach utilizing both objective and subjective ratings. We added more information how the different categories of intensity are defined:

l. 474-481. *“The intensity of exercise will be determined by a dual approach utilizing both objective and subjective ratings. Objective ratings will be guided by cut-off points derived from literature for heart rate reserve (%HRR), oxygen uptake reserve (%VO2R), maximal oxygen consumption (VO2max), maximal heart rate (%HRmax), repetition maximum (%RM). Subjective ratings will be guided by predefined cut-off points for METs (metabolic equivalents of tasks), ratings of perceived exertion (RPE), as well as specific exercise types classified by a Delphi survey conducted by the COST action Physagenet.”*

Reference:

American College of Sports Medicine. ACSM’s Guidelines for Exercise Testing and Prescription, 10th ed.; Wolters Kluwer: Alphen aan den Rijn, The Netherlands (2017). ISBN 9788578110796

l. 519. What are the parameters GRADE that will be assessing? Only the overall effect?

Response: No, we will assess all five different domains. We added further information to make that clear:

l. 580-582. *“The GRADE framework will be used to assess the evidence accumulated for each outcome overall and for the specific domains: 1) Risk of bias, 2) Imprecision, 3) Inconsistency, 4) Indirectness, 5) Publication bias.”*

l. 534. Where are these hypotheses stated?

Response: We agree. We did not formulate it as hypothesis but as research question. We included the hypothesis into the Introduction:

l. 270-284. *“We hypothesize that chronic exercise interventions differ in their efficacy in reducing overall depression severity and the different symptoms of depression in older adults across participants with different individual’s characteristics (i.e., age, sex, population, level of education at baseline, functional capacity at baseline, global cognition at baseline, depression severity at baseline) and across interventions with different exercise characteristics (i.e., frequency of exercise sessions, intensity of exercise, duration of exercise session, duration of intervention, type of exercise, cognitive demand of exercise, social interaction, use of behavioral change techniques, type of control group). Therefore, this protocol outlines a review aiming to investigate how the above-mentioned intervention characteristics and individual characteristics moderate the effects of chronic exercise on overall depression severity and the different symptoms of depression in older adults. The different symptoms of depression will be thereby defined using the criteria delineated by the DSM-5 [7] to make a diagnosis of depression: depressed mood, loss of interest/pleasure, weight loss or gain, insomnia or hypersomnia, psychomotor agitation or retardation, fatigue, feeling worthless or excessive/inappropriate guilt, decreased concentration, thoughts of death/suicide. Each of these symptoms will be used as secondary outcome in the present meta-analysis.”*

Reviewer #2: I would like to commend the authors on their work in presenting the protocol of a review article that focuses on exploring the impact of chronic exercise on depressive symptoms among older adults. The study's objective to delve into the effects of exercise on specific symptoms of depression, in addition to its overall severity, is a noteworthy contribution to the existing literature. The incorporation of various moderators and techniques to correct for publication bias adds sophistication to the protocol. While the manuscript is well-written and the protocol demonstrates a commendable level of detail, I do have a few suggestions that I believe could further enhance the overall quality of the study.

Response: We thank reviewer#2 for his/her comments that allowed us to improve the review protocol and address some important issues.

Rationale and Objective:

I appreciate the authors' intention to investigate different intervention characteristics as potential moderators. One aspect to consider is the distinction between supervised and unsupervised exercise treatments, which could be an important moderator. Incorporating supervision as a potential moderator could provide valuable insights. If the authors do not include supervision as moderator, please elaborate.

Response: We thank the reviewer for this valuable comment. We added the moderator variable “exercise supervision” as suggested:

l. 502-506. *“Exercise supervision during exercise practice categorized as 1) home based, 2) supervised by a professional, and 3) not specified. Within home-based programs, participants engage in the program independently, relying on written or video materials for guidance but lacking real-time feedback during exercise. In supervised programs, a professional coach or instructor provides guidance and feedback to participants throughout the sessions.”*

Method: Intervention:

The manuscript's title indicates a focus on chronic exercise, but the defined intervention duration of at least one week (with more than one exercise session) can be perceived as relatively short for a chronic exercise context. The focus on chronic exercise in the title is perhaps misleading.

Response: We appreciate the insightful suggestion of the reviewer and agree that two sessions is quite a short period of time and probably at the limit of the definition of chronic exercise. However, as we want to include as wide a range of intervention durations as possible to account for this as a moderator variable, we still think this is appropriate for our case. We expect smaller effect size for shorter program durations.

Method: Secondary Outcomes:

The authors' intent to assess the effects of chronic exercise on specific depression symptoms is commendable. However, it's worth considering the inclusion of symptoms like suicidal ideation and feelings of worthlessness (low self-esteem; e.g. Kandola et al., 2019). Incorporating these symptoms in the review could provide a more comprehensive overview of the subject matter.

Response: We agree with the reviewer that the secondary outcome low self-esteem is missing for our review. However, we already performed a preliminary overview of the RCTs on this topic to determine how many studies reported each outcome of interest. If several instruments were available to assess a given symptom, we defined an order determined by the validity and frequency of use of the measurement instrument. If not enough studies (less than 3) report a valid measure for a particular symptom, we will not assess that symptom as secondary outcome. Concerning suicidal ideation and feelings of worthlessness, we found very few RCTs measuring and reporting these outcomes (less than 3). We added further information to the manuscript:

l. 395-414. *“Secondary outcomes will include symptoms of depression delineated by the DSM-5 to make a diagnosis of depression. For the selection of the outcomes, we have adhered as closely as possible to each of the specific criteria, which are as follows: 1) Depressed mood most of the day, nearly every day, as indicated by either subjective report (e.g., feels sad, empty, hopeless) or observation made by others (e.g., appears tearful); 2) Markedly diminished interest or pleasure in all, or almost all, activities most of the day, nearly every day; 3) Significant weight loss when not dieting or weight gain (e.g., a change of more than 5% of body weight in a month) or decrease or increase in appetite nearly every day; 4) Psychomotor agitation or retardation nearly every day (observable by others, not merely subjective feelings of restlessness or being slowed down); 5) Insomnia or hypersomnia nearly every day; 6) Fatigue or loss of energy nearly every day; 7) Feelings of worthlessness or excessive or inappropriate guilt (which may be delusional) nearly every day (not merely self-reproach or guilt about being sick); 8) Diminished ability to think or concentrate, or indecisiveness, nearly every day; 9) Recurrent thoughts of death (not just fear of dying), recurrent suicidal ideation without a specific plan, or a suicide attempt or a specific plan for committing suicide. We have already completed a preliminary overview of RCTs on this topic to determine how many studies report each outcome of interest. If several instruments were available to assess a given symptom, we defined an order determined by the validity and frequency of use of the measurement instrument. An exception is the secondary outcome executive functions (see below). Here we will collect all reported measures. If not enough studies (less than three out of a total of approximately 110 studies) report a valid measure for a particular symptom, we will not assess that symptom as secondary outcome.“*

Additionally, I'd like to highlight the authors' approach to evaluating the symptom "significant weight loss when not dieting or weight gain" using BMI. Given that regular exercise might influence BMI changes, it's crucial to address the potential for misinterpretation, where weight changes could mistakenly signify loss of appetite or involuntary weight loss. The authors are encouraged to discuss how they plan to handle this potential issue.

Response: We agree with the reviewer and are aware of the potential for misinterpretation. We added a paragraph to the manuscript discussing this issue:

l. 444-451. *“We are aware that regular exercise might influence BMI changes and that this variable carries the potential for misinterpretation, as weight changes could mistakenly imply loss of appetite or unintended weight loss. Nonetheless, given the robust U-shaped association between overall depression severity and obesity status (i.e., underweight, normal weight, overweight / obese) (Noh et al., 2015), we consider this symptom to be of significant importance. It might be plausible that exercise leads to a normalization of BMI, with individuals with excessively high BMIs experiencing weight loss and those with excessively low BMIs gaining weight, rather than resulting in an overall reduction in BMI.”*

Method: Individual Characteristics:

The introduction's mention of physical fitness as a potential moderator of the exercise-depression relationship is noteworthy. I suggest considering the inclusion of baseline VO2max as an indicator of physical fitness, if such data is available, alongside the hand grip strength test and the 6-min walk test. Otherwise please indicate why you did not include VO2max as moderator.

Response: We agree with the reviewer that VO2 max at baseline could be a good indicator of functional capacity. However, in selecting the individual characteristic variables, we followed a similar approach as for the secondary outcomes, selecting only those where enough studies (more than 3) reported a valid measure for a given characteristic. We added further information to the manuscript to make that clear:

l. 536-540. *“We acknowledge the existence of additional suitable indicators for functional capacity, such as VO_2_max. However, in the process of selecting the individual characteristic variables, we followed a similar approach as for the secondary outcomes, selecting only those where enough studies (more than 3) reported a valid measure for a given characteristic.”*

Data Analysis:

The authors' choice to report the standardized mean difference from pre to post-treatment is appropriate. To further enrich the study, it might be valuable to explore and report any potential long-term effects of exercise, including whether participants maintained their exercise routines over time.

Response: We agree with the reviewer. This information is already included in our manuscript. We agree that it is easy to miss because of the table. But we believe it will be more visible after formatting:

l. 460-461. *“We will further consider changes in depression score and symptoms of depression from baseline to any available intermediary measurement or follow-up as secondary outcomes.”*
